# Lead thrombus under standard-dose edoxaban in a patient with normal to high creatinine clearance and protein S deficiency

**DOI:** 10.1186/s12959-021-00302-w

**Published:** 2021-07-17

**Authors:** Wei-Chieh Lee, Min-Ping Huang

**Affiliations:** 1 Institute of Clinical Medicine, College of Medicine, National Cheng Kung University, Tainan, Taiwan; 2 Department of Internal Medicine, Division of Cardiology, Kaohsiung Chang Gung Memorial Hospital, Chang Gung University College of Medicine, 123, Ta Pei Road, Niao Sung District, Kaohsiung City, 83301 Taiwan

**Keywords:** Lead thrombus, Edoxaban, Paroxysmal atrial fibrillation, Protein S deficiency

## Abstract

**Background:**

Non-vitamin K antagonist oral anticoagulants (NOACs) are as effective and safe as warfarin for thromboembolic prevention and treatment. The efficacy of NOACs lacks evidence from large and randomized studies in patients with inherited severe thrombophilia, including protein S deficiency. Further, some concerns still exist regarding the relative efficacy of edoxaban in preventing arterial thromboembolism in patients with normal to high creatinine clearance (CrCl). We present a case of a rare complication of lead thrombus under standard-dose edoxaban in a patient with protein S deficiency and supernormal renal function.

**Case presentation:**

A 65-year-old man experienced persistent chest tightness and a high level of D-dimer. Chest computed tomography (CT) showed a lead thrombus at the superior vena cava. He had a medical history including, paroxysmal atrial fibrillation (PAf), sick sinus syndrome after permanent pacemaker implantation, and transient ischemic attack. He received standard-dose edoxaban (60 mg daily) after PAf was diagnosed. His estimated CrCl was 98.6–102.1 mL/min. However, protein S deficiency (22.8%; normal range: 55–130%) was diagnosed. After switching to dabigatran (150 mg twice daily) for 3 months, the chest CT showed lead thrombus resolution and no symptoms were seen during the follow-up period.

**Conclusions:**

This case was a rare complication of lead thrombus in a protein S deficient patient with normal renal function receiving standard-dose edoxaban. Edoxaban efficacy is uncertain in patients with protein S deficiency, and intracardiac devices also increase the risk of thromboembolic events.

## Background

Non-vitamin K antagonist oral anticoagulants (NOACs) are as effective and safe as warfarin for thromboembolic prevention. In patients with inherited severe thrombophilia, including protein S deficiency, the efficacy of NOACs is still controversial. Some concerns still exist regarding the relative efficacy of edoxaban in preventing arterial thromboembolism in patients with normal to high creatinine clearance (CrCl). We present the case of a thromboembolic event (lead thrombus) that occurred from using standard-dose edoxaban in a patient with paroxysmal atrial fibrillation (PAf) and protein S deficiency but with normal renal function.

## Case presentation

**Fig. 1:**
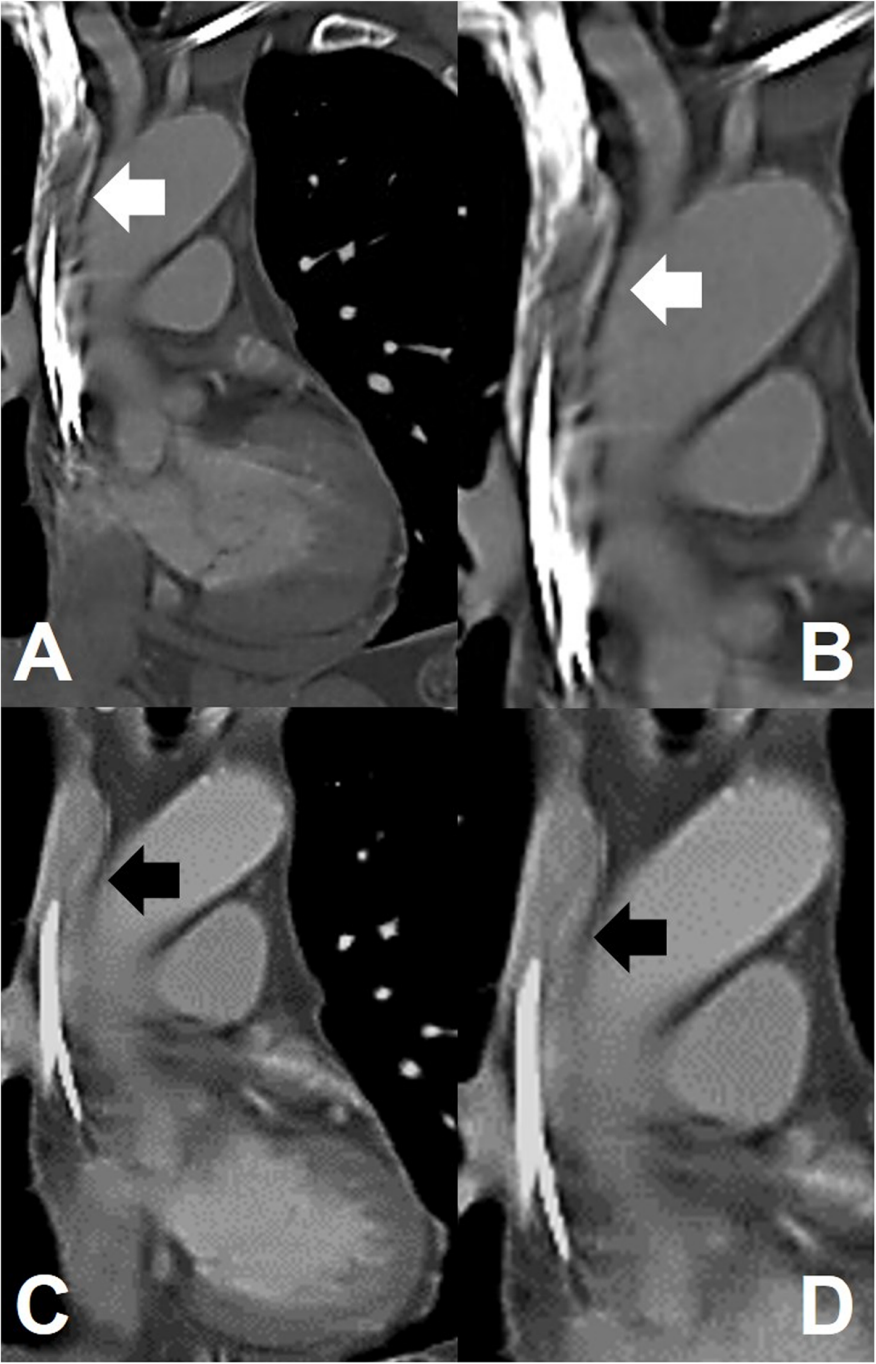
Chest computed tomography. **A**, **B**. A filling defect in the right internal jugular vein and superior vena cava was noted with highly suspected lead thrombosis coated on the pacemaker leads (white arrows). Follow-up chest computed tomography. **C**, **D**. No filling defect and no residual lead thrombus were noted (black arrows)

A 56-year-old man experienced chest tightness for hours, which gradually worsened for a day. Chest discomfort was more severe at the peak of inhalation. The tenderness was located at the central and upper chest wall, which radiated to the back. No cold sweating or compression was observed. He visited our emergency department for advanced evaluation and treatment. Electrocardiography showed normal pacemaker rhythm and no ST-T segment changes. Cardiac biomarker levels were normal, but a high D-dimer level (6.57 mg/L; normal range: < 0.5 mg/L) was noted. Emergency chest computed tomography (CT) revealed no pulmonary embolism but presented a filling defect in the right internal jugular vein and superior vena cava, which was highly suspected lead thrombosis (Fig. 1A, B; white arrows). Due to persistent symptoms, we did not suspect flow artefact or laminal effects.

**Fig. 2:**
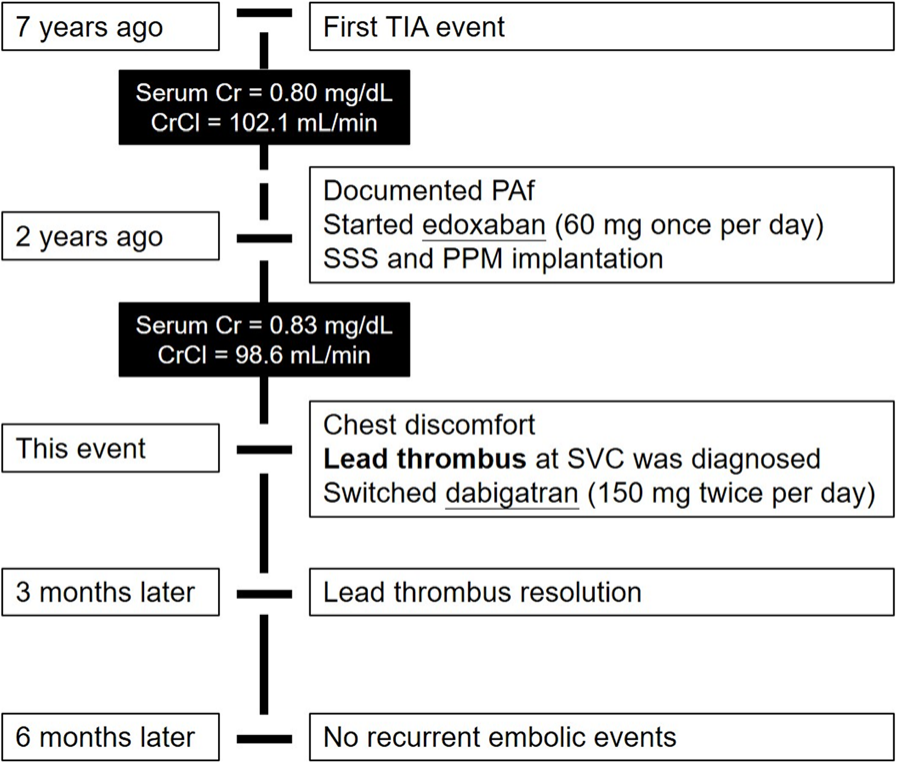
Perspective section about medical history. Abbreviations: TIA: transient ischemic attack; Cr: creatinine; CrCl: creatinine clearance; PAf: paroxysmal atrial fibrillation; SSS: sick sinus syndrome; PPM: permanent pacemaker; SVC: superior vena cava

The patient had a medical history of transient ischemic attack 7 years earlier. He also experienced dizziness and was diagnosed with PAf and sick sinus syndrome with long pauses (> 3 s) 2 years ago. After being diagnosed with PAf, he received standard-dose edoxaban (60 mg) daily for stroke prevention and received a transvenous permanent pacemaker for symptomatic bradycardia. CrCl estimated by the Cockcroft-Gault formula was supernormal to normal (98.6–102.1 mL/min). The burden of PAf was approximately 0.2–1% in pacemaker records after pacemaker implantation and the use of antiarrhythmic agents (amiodarone 100 mg daily). Intracardiac electrogram did not detect any atrial high rate in the last 3 months but noted protein S deficiency (22.8%; normal range: 55–130%). He did not receive p-glycoprotein or CYP3A4 inducers but did receive low-dose atorvastatin (10 mg daily). Then, 150 mg dabigatran twice daily was administered to resolve the thrombus and secondary prevention of thromboembolic events. Three months later, chest CT also showed no filling defect and no residual lead thrombus (Fig. 1C, D; black arrows). The patient denied recurrent chest discomfort during the half-year follow-up period. His medical history is listed in Fig. 2 and his biological characteristics are listed in Table 1.

**Table 1:** Summary of biological characteristics

Variable	Value	Normal range
D-dimer (mg/L)	6.57	< 0.5
Creatinine (mg/dL)	0.80–0.83	0.64–1.27
Estimated CrCl (mL/min)	102.12–98.57	
Anti-thrombin III (%)	99.2	75–125
Protein-S (%)	22.8	55–130
Protein-C (%)	77.3	70–140
C3 (mg/dL)	116.00	90–180
C4 (mg/dL)	21.30	10–40
ANA	negative	negative
A-DSDNA (WHOunit/mL)	< 40.5	< 92.6

*Abbreviation*: *CrCl* Creatinine clearance, *ANA* Antinuclear antibody, *A-DSDNA* Anti-double stranded DNA

## Discussion

This was a case of lead thrombus under standard-dose edoxaban and a diagnosis of protein S deficiency. The patient still had a medical history of PAf, but a low burden (0.2–1%) was noted in the intracardiac electrogram. No strong drug-drug interaction was noted, and the patient also presented good medical adherence. Low-dose atorvastatin did not affect the efficacy of edoxaban. Because he had supernormal to normal renal function, inadequate protection against thromboembolic events caused by edoxaban needed to be considered. Protein S deficiency and intracardiac devices also increased the risk of thromboembolism. Edoxaban was switched to dabigatran (150 mg twice daily), and chest CT 3 months later revealed resolution of the lead thrombus. The associated chest discomfort was also resolved. Therefore, there are three potential underlying reasons for the therapeutic failure in the present case: first, a high-risk patient with protein S deficiency; second, uncertain edoxaban efficacy in the presence of an intracardiac device; and third, uncertain edoxaban efficacy in a patient with normal renal function.

In large randomized controlled trials, NOACs have been shown to be as effective and safe as warfarin [1–4]. NOACs also reduced the composite end point of stroke (odds ratio = 0.65) and systemic embolic events (odds ratio = 0.85) when compared with warfarin [5]. Edoxaban acts as a direct factor Xa inhibitor and renal clearance accounts for approximately 50% of the total clearance of edoxaban [6]. There are some concerns regarding the relative efficacy of edoxaban in preventing arterial thromboembolism in patients with CrCl > 95 mL/min [7]. Such concerns are of particular interest considering the United States Food and Drug Administration label, which restricts its use in patients with CrCl > 95 mL/min because of concerns of decreased efficacy in preventing arterial thromboembolism compared with warfarin [8]. However, both doses of edoxaban have been associated with reduced risks of stroke and systemic embolism without decreased efficacy when standard-dose edoxaban has been used in the presence of higher CrCl (> 95 mL/min) in real-world practice [9].

Pacemaker implantation is complicated by major thromboembolic events in 0.6–3.5% of cases [10]. One case report stated pacemaker-associated thrombosis in ongoing therapy with reduced-dose edoxaban [11]. Few previous reports have discussed thromboembolic events in patients with supernormal or normal renal function. Two other case reports have presented lead thrombi under other anti-factor Xa inhibitors [12, 13]. NOACs have comparable efficacy to warfarin for treating and preventing venous thromboembolisms (VTEs) [14]. Natural anticoagulant deficiencies (protein C, protein S, or anti-thrombin), homozygous factor V Leiden (FVL), and prothrombin G20210A, or combined defects, result in a severe thrombophilic phenotype, occurring in approximately 5% of patients with idiopathic VTE [15]. However, few studies have focused on the efficacy of NOACs for the treatment of inherited severe thrombophilia. A case series reported lower efficacy of NOACs in patients with protein S deficiency [16], but it did not present any data regarding edoxaban. In two meta-analyses, the rates of VTE recurrence were both low and were comparable in patients with various thrombophilias receiving either treatment; therefore, NOACs are an appropriate treatment option in this population [14, 17].

In clinical practice, NOAC therapy does not require routine monitoring of NOAC levels for dose adjustment [18, 19]. However, NOAC level measurement needs to be considered if the patients present special situations including, (A) acute or chronic renal insufficiency; (B) thrombolytic therapy consideration; (C) clinically significant bleeding; (D) therapeutic failure with recurrent VTE; or (E) urgent or emergent invasive procedures [20, 21]. Additionally, there are other scenarios where NOAC levels could be useful such as in patients with extreme body weights, the presence of interacting medications, and confirmation of chronic anticoagulation following the initial loading period [22]. Unfortunately, NOAC level measurements were not available in our hospital, so we did not know the efficacy of edoxaban in this patient with protein S deficiency, an intracardiac device, and normal renal function. Therefore, we switched to the direct thrombin inhibitor dabigatran to solve the therapeutic failure of edoxaban. Fortunately, the lead thrombus resolved, and no recurrent thromboembolic events happened in the follow-up period.

## Conclusions

This case was a rare complication of lead thrombus in a patient with normal renal function receiving standard-dose edoxaban. Eoxaban inefficacy can be suspected in patients with protein S deficiency, and intracardiac devices also increase the risk of thromboembolic events.

## Data Availability

Data available on request from the authors.
